# PPAR Gamma: Coordinating Metabolic and Immune Contributions to Female Fertility

**DOI:** 10.1155/2008/243791

**Published:** 2008-01-03

**Authors:** Cadence E. Minge, Rebecca L. Robker, Robert J. Norman

**Affiliations:** Research Centre for Reproductive Health, School of Paediatrics and Reproductive Health, The University of Adelaide, South Australia 5005, Australia

## Abstract

Peroxisome proliferator-activated receptor gamma (PPARG) regulates cellular functions such as adipogenesis and immune cell activation. However, new information has indicated additional roles of PPARG directing the cyclic changes that occur within ovarian tissue of female mammals, including those that facilitate the release of oocytes each estrous cycle. In addition to ovarian PPARG expression and function, many PPARG actions within adipocytes and macrophages have additional direct and indirect implications for ovarian function and female fertility. This encompasses the regulation of lipid uptake and transport, insulin sensitivity, glucose metabolism, and the regulation of inflammatory mediator synthesis and release. This review discusses the developing links between PPARG activity and female reproductive function, and highlights several mechanisms that may facilitate such a relationship.

## 1. INTRODUCTION

Since its initial identification in the early 1990's, peroxisome proliferator-activated receptor gamma (PPARG) has been primarily recognised as a regulator of cellular functions such as adipogenesis and immune cell activation. However, some recent reviews have discussed additional roles of PPARG directing the cyclic changes that occur within ovarian tissue of female mammals, including those that facilitate the release of oocytes each estrous cycle [1–4]. In addition to ovarian PPARG expression and function, many PPARG actions within adipocytes and macrophages have additional direct and indirect implications for ovarian function and female fertility. For instance, PPARG, through activation by thiazolidinediones (TZDs), is known to regulate the metabolism of lipids, providing both self-regulatory PPARG transcriptional mechanisms, and stimulating an increase in adipogenesis. Whilst the net volume of adipose tissue carried within an individual can influence reproductive potential, genes associated with lipid metabolism are also important for ovarian cells directly. As a result, PPARG has the potential to influence the cellular operations of follicles containing oocytes and, consequently, the health of those oocytes released. Likewise, the PPARG regulation of insulin sensitivity, downstream signalling pathways, and ultimately glucose uptake are likely to be also vitally important for normal ovarian function and overall female fertility.

Similarly, PPARG regulation of macrophage function has been addressed in vitro and within the context of the adipose tissue for many years; but appropriate activity of resident immune cells is also a prerequisite for normal ovarian function, as they are required for tissue remodelling facilitating ovulation, luteinization, and luteolysis [5]. Therefore, not only are adipose/circulating macrophage-sourced inflammatory mediators sensed by ovarian cells, but these mediators, when produced locally by the ovary, may influence the ovarian function in an autocrine fashion.

This review aims to provide evidence for how PPARG-regulated pathways influence the female's ability to produce healthy, developmentally competent oocytes. This is impacted by cellular function operating primarily at the local ovarian level, either directly acting upon the oocyte itself, or influencing the supporting ovarian cells that supply the oocyte with hormonal signals and nutrients. In addition, signals from extraovarian tissues, in particular adipose tissue and the circulating and/or resident immune cells, also exert powerful influences over the normal function of the ovary.

These concepts of overlapping influence on female fertility are particularly important when we consider conditions of reduced and impaired fertility such as polycystic ovary syndrome (PCOS), as well as reduction of reproductive function associated with excessive bodyweight and insulin resistance. In these situations, profound dysregulation of both metabolic and immune signalling pathways exacerbate ovarian perturbations, which are often successfully treated with administration of PPARG-activating pharmaceuticals.

## 2. PPARG GENE EXPRESSION

Successful mammalian reproduction requires a female body adequately, but not excessively, nourished, equipped to produce healthy eggs and to supply a growing fetus with sufficient energy. In this way, many tissues within the female body are able to influence the level of fertility. The extent of PPARG expression and its temporal regulation within these tissues can provide an interesting insight into the role of PPARG in female fertility.

### 2.1. Ovarian PPARG

**Figure 1:**
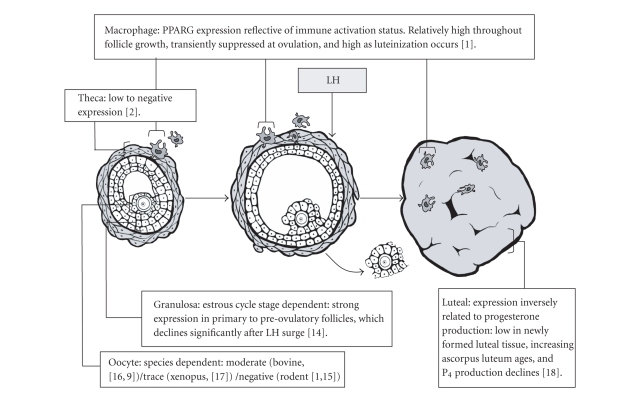
Overview of PPARG expression by specific ovarian cell types, as follicular development progresses from early antral and preovulatory follicle to postovulatory corpus luteum.

Within the ovary, processes that are modulated by the PPAR superfamily, particularly PPARG, are among the most critical to normal ovarian function (Figure 1). Steroidogenesis, tissue remodelling, angiogenesis, lipid metabolism, immune cell activation, and production of proinflammatory mediators are all, to some extent, controlled by the presence and activity of the PPAR nuclear receptors. All three PPAR isotypes have been identified in the ovary of many species including the rat [2, 6], mouse [7], pig [8], sheep [9], cow [10, 11], and human [12, 13]. Localisation of these nuclear receptors has been established by both in situ hybridisation and immunohistochemistry [6]. Transcripts for PPAR alpha (PPARA) have been identified in immune cells and cells in the theca and stroma, whilst PPAR delta (PPARD) is found across all ovarian compartments [2]. Ovarian expression of both PPARA and PPARD is relatively stable across the ovulatory cycle, which suggests these isotypes are likely involved in regulating basal ovarian functions. PPARG is expressed strongly in the granulosa cells (primarily responsible for both estradiol production and the regulation of follicular fluid content), and lessstrongly in the thecal-region (site of androgen precursor production for granulosa estradiol synthesis) and luteal cells (postovulatory progesterone production) in the ovaries of rodents and ruminants [1, 2, 9, 14]. PPARG is detected earlyin folliculogenesis, and in contrast to PPARA and PPARD isotypes, its expression is dynamic, increasing until the large folliclestage [9], followed by downregulation in response to the LH surge [2].

Within the oocyte itself, PPARG expression seems to be dependent upon species, as moderate expression has been reported in ruminants [16], trace levels in Xenopus oocytes [17], and undetectable expression in rodents [1, 18]. It has not yet been investigated within the human oocyte.

### 2.2. Extraovarian PPARG

The highest level of mammalian PPARG expression is found within adipose tissue [19, 20], and activation of this adipose PPARG is sufficient [21] and essential to induce adipogenesis [22, 23]. Adiposity is also a key regulator of female fertility, affecting multiple aspects of the reproductive axis in women [24, 25]. Many of the adipocyte-sourced factors that are under PPARG control, such as the production of non-esterified free fatty acids, have widespread effects including ovarian targets [26–28]. In addition, any activation of adipose PPARG that may influence the amount and activity of adipocytes/adipokines can subsequently impact upon reproductive potential [29].

Both the ovary and adipose tissue are comprised of a considerable proportion of immune cells, in particular macrophages. Macrophages recruited into tissues are an important source of many inflammatory mediators that have functions both locally and systemically. Within the ovary, macrophage contribution to the pool of functional PPARG has been assessed [1]. TZD treatment has also been found to affect adipose-recruited macrophages, by increasing the rate of apoptosis, providing a subsequent reduction in the number of proinflammatory cytokine-producing cells [30]. Improvements to the chronically inflamed profile of women with PCOS may well go some way in explaining the beneficial systemic effects of PPARG activation in such patients (see Sections 3.2 and 4.4).

### 2.3. Mutations in PPARG negatively influence female fertility

**Figure 2:**
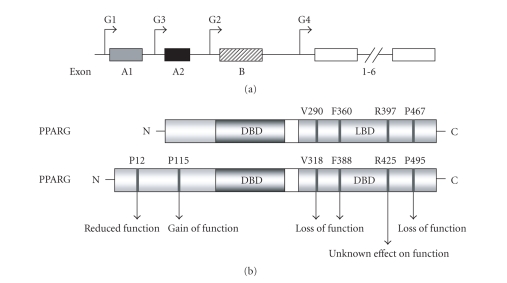
(a) The genomic structure of the $5^{\prime }$ end of the human PPARG gene. Exons 1-6 are common. Exons A1 and A2 are untranslated, and exon B is translated, giving rise to two different proteins corresponding to the G1 or G2 transcripts. (b) The domain structure of PPARG1 and G2 isoforms with the positioning of mutations or polymorphisms resulting in substituted amino acid residues, and altered protein functions. DBD, DNA-binding domain; LBD, Ligand-binding domain. (Figure adapted from Sundvold and Lien[33], Tsai and Maeda [37], and Stumvoll and Häring [38]).

The PPARG gene contains 9 exons, and spans more than 100 kb [31] (Figure 2(a)). There are at least 4 isoforms of PPARG, resulting from the use of different initiator methionines [31–33], which are believed to be involved in regulated gene expression in specific cells and tissues. PPARG1, expressed utilizing the untranslated exons A1 and A2, is 477 amino acids long, and is expressed at low levels in many tissues [34]. PPARG2 contains the translated exon B, and as a result is 28 amino acids longer than PPARG1 [35]. This isotype is expressed selectively in white adipose tissue, colonic epithelium, and macrophages [36]. PPARG3, which contains only exon A2, is found only in the large intestine and macrophages [31]. PPARG4 is limited to exon 1-6 common to all isotypes [33]. There have been numerous studies into the effects of genetic variability of PPARG gene sequence and expression, in both rodent models and human patients (Figure 2(b), Table 1).

Work initiated in rodent knockout models revealed that total PPARG^−/−^ mutants display two independent lethal phases [23]. Firstly, PPARG deficiency interferes with terminal differentiation of the trophoblast cells and with placental vascularization, leading to myocardial thinning, and death by embryonic day 10. When PPARG null embryos are provided with a wild-type placenta, this cardiac defect was corrected permitting delivery, although postnatal pathologies (including multiple haemorrhages and lipodystrophy) resulted in lethality. To circumvent such restrictions, the Cre-loxP system can be applied, where Cre recombinase was under the control of the whey acidic protein (WAP) or mouse mammary tumour virus (MMTV) promoters. This causes PPARG gene deletions specific to secretory and hematopoietic tissues (alveolar epithelial cells of mammary tissue, salivary gland cells, oocytes, granulosa cells, megakaryocytes, and B- and T-cells) [50]. The results of this study revealed an important PPARG role in fertility: although the mutant mice appeared to ovulate normally, they exhibited reduced progesterone secretion as well as impaired implantation. Interestingly, fertility is affected even when the lesion in PPARG expression is restricted to extraovarian sites, as homozygote matings of PPAR^hyp/hyp^ mutants, lacking PPARG expression in white and brown adipose tissue, liver, and muscle, had reduced litter size [40].

Examinations of naturally occurring human polymorphisms have focussed on susceptibility to Type II diabetes, insulin sensitivity, and obesity, and to date at least seven polymorphisms within the PPARG gene have been described.

The Pro12Ala polymorphism is located in exon2, and is only translated within the adipose tissue-, macrophage-, and colonic epithelium-specific PPARG2 isotype. The Pro12 allele is carried by approximately 85% of certain regional populations [51], and a single nucleotide mutation (C→G) leads to the substitution of an Ala amino acid [41]. PPARG protein produced by the Ala12 allele shows reduced in vitro affinity for PPAR response elements (PPREs) in target gene proximal promoters, and subsequently has reduced PPARG transactivation [41]. This PPARG SNP was extensively studied, following initial reports that it was strongly associated with bodyweight and insulin sensitivity [41], and the effect of the Ala12 mutation on PCOS symptoms has been closely studied, although some specific conclusions are difficult to reach. There are conflicting reports regarding the effect of this allele on BMI: either linked with increased BMI [45, 52–55], lower BMI [41, 56–59], or not associated at all [45, 60–64]. Current assumptions are that differential environmentalinteractions between populations can modify the function of this polymorphism. However, the relationship between Pro12Ala and insulin sensitivity appears more conclusive. Populations of women positive for Ala12 and PCOS have lower fasting insulin, reduced measures of systemic insulin resistance, lower insulin secretion, and lower hirsutism scores than women without the allele [54, 65, 66]. Consequently, the frequency of this allele is much lower in groups categorised as PCOS [54, 65, 67]. It appears that the Pro12Ala polymorphism of the PPARG gene may be a modifier of insulin resistance in women with PCOS, which can have a profound influence on fertility (see Section 4.1).

Another PPARG2-specific polymorphism is the rare Pro115Gln substitution in exon 3 that results in permanent, ligand independent activation [44]. This induces excessive adipocyte differentiation, and as a result the 4 individuals known to carry this (nonfamilial) SNP suffer extreme obesity [44], although present with only moderate metabolic complications including Type 2 Diabetes. The reproductive implications of hyperactive PPARG2 have not been addressed in these subjects.

All other reported polymorphisms are located in regions common to both PPARG1 and PPARG2. The His447His polymorphisms resulting from a C to T substitution at nucleotide 1431 in exon 6 is a silent polymorphism that encodes histidine with either allele [55]. Also referred to as the C161T polymorphism, it is proposed that this substitution may modulate expression of PPARG by altering mRNA processing or translation, leading to increased adipocyte differentiation. Subsequently, carriers of the T allele have elevated BMI. The T allele is also more common in women with PCOS compared to non-PCOS BMI-matched controls [45], and therefore has suspected involvement in the high incidence of obesity in PCOS population. However, both PCOS subjects and controls with T allele appear to be protected from other complicating symptoms of obesity, having better insulin sensitivity in addition to lower circulating testosterone.

The remaining polymorphisms are all extremely rare and restricted to single families.

Reported by Barroso et al. [46] and Savage et al. [47], there is a PPARG1 Pro467Leu substitution in the region required for ligand-dependent transactivation (PPARG2 residue 495) which results in impaired coactivator recruitment and downstream transactivation. This mutation also inhibits basal gene activity and has been found within 4 members of a single family spanning 3 generations. Medical histories reveal that in addition to lipodystrophy and hypertension (both frequently associated with PPARG mutations), the female carrier also experienced oligomenorrhea and hirsutism, and required ART intervention to conceive. This, and a subsequent spontaneously conceived pregnancy were both complicated with severe pre-eclampsia. Treatment of the male carrier with rosiglitazone (8 mg/day) was found to normalise chronic hyperglycaemia after 6 months, suggesting that the mutant PPARG protein is still able to be activated by exogenously sourced ligands, indicating the phenotypic profile of these subjects results from abnormal basal and endogenously activated PPARG activity.

Also identified by this study was a similarly positioned PPARG1 Val290Met mutation (PPARG2 residue 318) in a single female individual. This mutation results in a profound loss of PPARG function evidenced by both in vitro reporter gene activity, and in vivo response to rosiglitazone. Experiencing comparable metabolic complications to subjects with the Pro467Leu substitution, this individual also reported primary amenorrhoea, hirsutism, and acanthosis nigricans. Implications of these gynaecological and endocrine aberrations relating to conception and pregnancy have not been reported.

Another loss-of-function mutation is the phenylalanine to leucine substitution at position 388 (reported with respect to PPARG2, the substitution corresponds to residue 360 in PPARG1) found in 4 individuals from 3 generation of a single family [48]. Despite the reduction in normal PPARG function, concurrent treatment of one individual with both metformin and rosiglitazone (8 mg daily) provided effective glycemic control. Two of the affected individuals were female (46-year-old mother and her 22-year-old daughter), with the older individual experiencing a history of irregular menses and polycystic ovarian disease, eventually treated with bilateral salpingo-oophorectomy. At the time of study, the daughter did not have any significant medical problems (other than diet-controlled hyperinsulinemia and mild type IV hyperlipoproteinia), with regular menses and no polycystic ovarian pathology observed.

A heterozygous arginine to cysteine mutation at position 397 in PPARG1 (corresponding to residue 425 in PPARG2) was identified in a 64-year-old woman in 2002 by Argarwal and Garg [49]. Although the effect on PPARG functionality has not been explicitly described, but the mutation lies in a region of the protein that forms a salt-bridge, and as a result, the mutated form may lack proper protein configuration. The Arg397Cys substitution was associated with lipodystrophy, elevated triglycerides, and early-onset Type 2 Diabetes. In addition, although pregnancy was never sought, moderate hirsutism as well as a history of delayed menarche (age 18) and subsequently irregular menstrual cycles were reported.

Overall, these studies demonstrate that PPARG precisely controls various aspect of systemic metabolism in humans. As female fertility is also disrupted in a significant number of these patients, it is likely that PPARG regulates female reproduction either directly, by intrinsic actions within reproductive organs such as the ovary, or indirectly via the myriad effects on metabolic tissues such as adipose and liver. To better define links between the metabolic and reproductive consequences observed in so many of these PPARG mutations, it would be interesting to recapitulate, in a tissue-specific manner, some of these PPARG genetic aberrations in mice.

## 3. LIGANDS

Together with expression of PPARG itself, availability of ligands is a primary regulating factor determining the ability of PPARG to influence target gene expression. Ligands can be produced endogenously, providing physiological significance, or sourced exogenously, as therapeutic factors given to target specific metabolic and reproductive symptoms.

### 3.1. Endogenous ligands: physiological function of PPARG

All PPARs bind and are activated by naturally occurring fatty acids and their metabolites [68], thus acting as fatty acid-activated receptors that function as key regulators of glucose and cholesterol metabolism. The precise nature of endogenous PPARG ligand binding and activation remains poorly defined and more research is needed in this area. However, the potential for important physiological ovarian PPARG activation is considerable, as many natural ligands have been shown to be present within the ovary, and produced locally by ovarian cells. Included in this list are *ω*3- and *ω*6-polyunsaturated fatty acids (PUFAs) such as the essential fatty acids linoleic acid, linolenic acid, arachidonic acid, and eicosapentanoic acid ([69] and reviewed [34]). Additional PPARG agonists such as prostaglandin metabolites of these substances and immunologically-derived eicosanoids are also produced within the ovarian environment in a hormonally regulated manner, with elevated production as ovulation progresses [70–73]. It is possible that PPARG may have a role in the feed-forward production of eicosanoid ligands, based on identification of a PPRE in the prostaglandin-endoperoxide synthase 2 (a.k.a. COX-2) promoter [74], which would facilitate amplified production of pro-ovulatory prostaglandins.

### 3.2. Exogenous ligands: therapeutic application of PPARG activation

As information emerges regarding the endogenous roles for naturally activated PPARG within the ovarian follicular environment, other evidence of PPARG involvement with ovarian function comes from reports utilising synthetic PPARG ligands, specifically, administration of TZDs to women diagnosed with PCOS (Table 2).

PCOS is the leading cause of infertility and menstrual irregularities in women of reproductive age and is characterised by chronic hyperandrogenic anovulation [90]. This is thought to be due, in general, to hypothalamic-pituitary axis dysregulation causing elevated basal LH levels that overstimulate cells of the theca interna [91]. Insulin resistance also appears to contribute to the syndrome in many instances [92], as the pituitary responds to elevated plasma levels of insulin to augment LH release [91].

The potential merits of applying TZDs to improve reproductive outcomes in infertile PCOS women was first demonstrated by Azziz et al. [93]. Since then, treatment of PCOS patients with the TZDs rosiglitazone or pioglitazone have been shown to not only improve insulin action in peripheral tissues, attenuate hyperinsulinemia, and lower circulating levels of lipids [92, 94], but also to improve a range of reproductive outcomes particularly circulating sex hormone levels, and ovulation rate [77, 88, 90, 95–97] (see recent reports summarised in Table 2).

The beneficial effects of TZDs on ovarian PCOS symptoms were first attributed to improvements in defective insulin action and secretion [92]. However, actions upon various ovarian cells directly illustrated both in vitro [1, 9, 14, 98, 99] and in vivo [100, 101] confirms a direct interaction between these compounds and ovarian PPARG.

Particular focus has been directed upon the effect of PPARG activation on the synthesis of ovarian steroid hormones and the expression of many rate-limiting steroidogenic enzymes has been investigated.

1. Steroidogenic acute regulatory protein (StAR): facilitates that rapid mobilization of cholesterol for initial catalysis to pregnenolone by the P450-side chain cleavage enzyme located within the mitochondria [102]. It has been recently reported that both rosiglitazone and pioglitazone significantly up regulate StAR protein synthesis by human granulosa cells in vitro [103].
2. 3*β*-hydroxysteroid dehydrogenase (3*β*-HSD): catalyses the conversion of pregnenolone to progesterone by luteal cells [104]. Work on porcine granulosa cells has found that troglitazone competitively inhibits 3*β*-HSD enzyme activity within these cells [99].
3. Steroid 17-alpha-hydroxylase (P450c17): converts progesterone to androgen within ovarian theca cells [105]. Conflicting reports have arisen regarding the effect of TZDs on the expression and activity of this enzyme, many of which may be artefacts of various culture conditions. P450c17 mRNA production has been found to increase following porcine thecal cell exposure to TZDs [8], whilst other reports indicate suppression of enzymatic expression and/or activity in primary porcine thecal cells or human cell lines [106–108].
4. P450 aromatase (P450arom): aromatises androgen precursor to estradiol, and is expressed by ovarian granulosa [109] and luteal [110] cells. Although there is no correlation between the expression of the P450arom enzyme and PPARG itself during folliculogenesis, many reports have described the down-regulation of P450arom following TZD exposure in human ovarian cell cultures [13, 111–113].

Taken together, these findings provide strong evidence for the direct effect of TZD administration and PPARG activation on ovarian hormonal synthesis and secretion. Specifically the following.

1. Androgen: TZDs found to inhibit LH- and insulin-stimulated androgen biosynthesis by purified porcine thecal [108], and mixed human ovarian [98] cells. They have also been found to reduce plasma testosterone levels in women with PCOS [76, 77, 81–84, 86, 89, 91, 97, 114, 115].
2. Estrogen: While it is accepted that TZDs indeed influence estrogen secretion, estrogenic responses to TZDs appear to be dependent on confounding factors such as species, age, and endocrine setting. For instance, TZDs have been found to increase estradiol secretion [2], and decrease estradiol production [116]. PPARG activation by TZDs and phthalate toxins are believed to mediate the antiestrogenic effects of these agents in cultured rat granulosa cells [116], and TZDs have also been found to suppress stimulated estradiol secretion in human granulosa cell cultures [98].
3. Progesterone: As for estrogen, progesterone responses to PPARG activation via natural or endogenous ligands are unclear, and are probably regulated by species, and stage of folliculogenesis. Most publications investigating a range of species, including primary bovine, ovine, porcine, or rodent cell cultures, report increases in progesterone secretion following administration of PPARG activators in vitro [2, 8–10], whilst some others suggest inhibition of stimulated progesterone secretion by porcine granulosa cells [14].

The net influence of TZD treatment on ovarian PPARG activation and subsequent steroidogenesis in vivo remains poorly defined across all species investigated. The most conclusive evidence for an advantageous outcome on hormonal (specifically androgen) profile following treatment is observed in women with PCOS, as overviewed in Table 2. As a result, increasing attention may be paid towards the application of these drugs in such conditions of significant hormonal perturbations.

Rosiglitazone and pioglitazone are currently listed as a Pregnancy Category C drug (i.e., not tested for use during human pregnancy), and some side effects of TZD administration, such as weight gain, fluid retention (reviewed in [117]), and possible bone demineralisation [118], preclude their widespread use during pregnancy. However, in vitro treatment of 2 cell mouse embryos, or in vivo treatment of pregnant mice with rosiglitazone was not found to impact upon normal blastocyst development, or litter rates and sizes [119]. In situations where conception has occurred following TZD treatment for PCOS, no adverse fetal outcomes have been observed [88, 96, 120]. Also in a recent study, examining tissue obtained from women with scheduled pregnancy terminations, it was found that placental transfer of maternally administered rosiglitazone to fetal tissues is minimal in the first 10 weeks of pregnancy [121].

## 4. MECHANISMS: PPARG REGULATION OF METABOLIC AND IMMUNE FACTORS INFLUENCING FEMALE FERTILITY

PPARG is known to regulate many pathways involving insulin sensitivity, glucose metabolism, adipokine signalling, lipid uptake and metabolism, and secretion of inflammatory mediators. As a result, PPARG is being revealed as a key mediator of the fundamental metabolic and immune contributions that are required for normal female fertility.

### 4.1. Insulin sensitivity

Normal insulin sensitivity and subsequently efficient metabolism of glucose are essential for healthy reproduction in the female. Conditions of hyperinsulinemia can interfere with normal ovarian cell function or be indirectly associated with other hormonal conditions detrimental to optimal fertility [122–124]. Also, exposure to high levels of glucose can have a deleterious effect on the oocyte [125, 126]. By normalising peripheral insulin signalling, PPARG activation can circumvent many of these adverse effects of hyperinsulinemia, as well as those detrimental outcome associated with persistently elevated blood glucose levels.

The genetic studies detailed above, and the pharmacokinetics of TZD treatment improving insulin sensitivity are both consistent with a direct role for PPARG in the regulation of cellular insulin utilization. Despite this, it remains to be determined exactly how TZD treatment and subsequent PPARG activation impacts gene expression directly related to insulin signalling and glucose uptake (through genes such as the insulin receptor (IR), IR-substrates, and glucose transporters), as a range of conflicting results have emerged. Suggested mechanisms include increases in glucosetransport protein 4 (GLUT4), stimulation of phosphatidyl-3-kinase andmodified phosphorylation of insulin receptor substrates [127–133]. In addition, it is well accepted that activation of PPARG does improve not only basal hepatic glucose secretion, but also peripheral insulin-stimulated glucose uptake, potentially indirectly via reduction of FFA, TNF*α*, plasminogen activator inhibitor-1, and other autocrine/endocrine signalling molecules which otherwise interfere with efficient insulin signalling (reviewed in [134]). In this way, PPARG activation may improve female infertility exacerbated by obesity and insulin resistance [25, 135–141].

New reports are also describing some of the first investigations into the ovarian-specific responses to TZD that facilitate insulin sensitivity in this tissue. The work of Seto-Young et al. [103] has shown that ovarian cells directly respond to TZDs to increase transcription of insulin signalling components including IR alpha and beta subunits and IRS-1, which would subsequently provide more efficient signal transduction and cellular response to insulin.

### 4.2. Adipokines: leptin and adiponectin

Produced primarily by adipose tissue, leptin and adiponectin are “adipokines” with contrasting actions on insulin sensitivity. Whilst other adipokines such as visfatin and retinol-binding protein 4 (RBP-4) are also linked with insulin sensitivity [142, 143] and the incidence of PCOS [144, 145], leptin and adiponectin are of particular interest to those investigating female reproduction as it is known their presence can be detected by ovarian cells which express leptin and adiponectin receptors. In addition, although only the adiponectin promoter has been shown to contain a PPRE [146], transcriptional activity of both leptin and adiponectin genes is known to be decreased and increased, respectively, in the presence of PPARG-activating ligands [147–151]. In this way, they can operate as secondary messengers of signals initiated by PPARG activation.

Leptin receptors are present in the granulosa and thecal layers of the ovary [152, 153], and have been shown to be cyclically regulated [154]. Leptin appears to influence ovarian gonadotropin and steroid secretion [152, 153, 155], and affect oocyte quality and developmental potential [156, 157].

Adiponectin receptors AdipoR1 and AdipoR2 are also both expressed by ovarian tissue [158] and adiponectin itself has been identified within the follicular fluid of developed follicles in similar concentrations to that observed in the serum [159]. Adiponectin appears to be involved in many processes including those essential for ovulation, such as induction of COX-2 and prostaglandin E synthase expression in ovarian granulosa cells [159].

As the entire range of leptin and adiponectin effects on ovarian cellular functions, including the outcomes of PPARG activation (including enhancement of insulin sensitivity), are gradually established, it is likely we will find that the improvements to reproductive profiles and ovarian function of sub-fertile or infertile women treated with TZDs are mediated, at least in part, through modulation of these two adipokines.

### 4.3. Lipid uptake: CD36 and SCARB1

PPARG has a critical role in the regulation of adipocyte differentiation [94]. Among the best characterised PPARG target genes are those involved in lipid metabolism, including phosphoenolpyruvate carboxykinase [160], lipoprotein lipase [161], fatty acid binding protein [162, 163], and CD36 and SCARB1 [164, 165]. CD36 and SCARB1, class B scavenger receptors that mediate the endocytosis or selective cholesterol uptake from oxLDL and HDL lipoproteins, are also both strongly expressed by the ovary. The CD36 antigenis highly expressed by granulosa cells of preantral and earlyantral follicles, with moderatestaining also evident in the vascular thecal layers [166].In this context, CD36 has been reported as a facilitator of thrombospondins-1 and -2 activities [166, 167], influencing cell adhesion, wound healing, and angiogenesis [168, 169]; important components of the tissue and cellular changes that occur during the ovarian cycle. CD36 is upregulated following activation of PPARG in macrophages [164, 170], and a summary of PPARG control of gene expression [171] suggested this might act as a positive feedback mechanism, such that more potential PPARG ligands can be imported, enhancing expression of both PPARG and CD36.

Ovarian SCARB1 expression appears to be strongly associated with HDL-cholesterol ester requirement for production of androgen for aromatase-mediated conversion to estradiol by the granulosa cells, and progesterone synthesis by luteal cells. Thecal cells consistently express high levels of SCARB1 at all stages of both healthy and atretic follicle development [172], and high expression is also found within luteal structures [173].

In these respects, PPARG activation may have profound influence on ovarian function through the regulation of these genes or others regulating lipid metabolism, by affecting availability of substrate for hormone synthesis, and the remodelling of tissue structures required for oocyte release, luteinization, and luteolysis.

### 4.4. Suppression of chronic inflammation

An important role for PPARG is the suppression of immune cell synthesis and secretion of proinflammatory mediators [174–182] (reviewed [183–185]). The role of the immune system in female fertility is critical, both systemically, and locally at the ovarian level.

In addition, there are also interesting correlations between the development of adiposity, insulin resistance and, chronic inflammation. Increased serum concentrations of TNF, NO, and IL-6 are strongly associated with obesity [186, 187], and proinflammatory cytokines sourced from adipose tissue including TNF, and IL-6 are among several important factors that participate in the development of insulin resistance and type 2 diabetes mellitus [188–191]. Interestingly, together with central adiposity and insulin resistance, we also find aspects of systemic inflammation independently associated with impaired female fertility and PCOS [192, 193]. PPARG is implicated in improvements to the systemic inflammation observed in obese and insulin resistant individuals treated with TZDs. These studies describe reductions in serum C-reactive protein, IL-6, and soluble TNF receptor 2 [194–198]. Other studies investigating the chronically inflamed profile of PCOS patients support these findings, reporting that in addition to restoring menstrual cyclicity and improving markers of hyperandrogensism, TZD treatment is able to lower circulating C-reactive protein levels and the number of circulating leukocytes [75, 85].

### 4.5. Ovarian macrophages

Macrophages, dendritic cells, lymphocytes, and neutrophils have unique roles in the context of ovarian physiology, and are essential for the normal regulation of ovulation and control of the reproductive cycle [5, 199–201]. Macrophage distribution and numbers within the ovary varies across the cycle, influenced by gonadotrophins and ovarian steroidogenic hormones. Resident macrophages are present in the theca and stroma of the ovary during the late stages of folliculogenesis [202]. Once the LH surge commences prior to ovulation, there is a massive recruitment of new leukocytes from the circulation into the theca of the preovulatory follicle [202, 203], where they function to release proinflammatory cytokines and mediators assisting the breakdown of the ovarian epithelium at ovulation. Their presence persists until after ovulation, further increasing in number in the developing and regressing corpus luteum [204].

Ovarian macrophages maintain high levels of PPARG transcript expression until a significant reduction in response to the proovulatory LH surge [1]. Immediately following ovulation, expression is restored to high preovulatory levels [1]. In vitro treatment of purified ovarian macrophages with the TZD troglitazone has been shown to significantly alter proinflammatory gene expression [1]. Specifically, these cells respond to TZD exposure by significantly suppressing mRNA production of NOS2 (or inducible Nitric Oxide Synthase, iNOS), the enzyme that catalyses the reaction producing the potent vasodilator, nitric oxide (NO). In the human, NO seems to direct follicular selection and maturation [205], and application of this NO property to IVF patients, deemed “poor responders”, has been found to increase the number of oocytes retrieved [206]. This is an indication that recruited and specialized ovarian macrophages can potentially respond directly to TZDs administered systemically, and can regulate the availability of ovulatory mediators. Such responses parallel the anti-inflammatory effects of PPARG activation in nonovarian-activated macrophages [171], but was here found to be specific to macrophages closely associated with the ovarian environment (distinct to those located in the peritoneal cavity for instance). This illustrates the unique influence of the ovarian milieu on normal PPARG function and effects.

## 5. CONCLUSIONS

**Figure 3:**
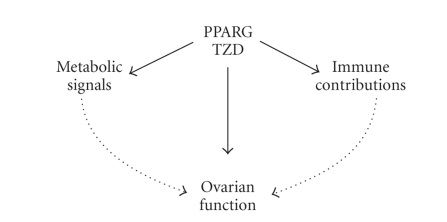
Schematic summarising the developing concept of PPARG influence on ovarian function and female fertility. PPARG is able to strongly influence the activity of ovarian cells directly, in particular steroidogenesis and tissue remodelling. In addition, PPARG can further influence ovarian function via regulation of external metabolic signals and immune cell contributions.

Many diverse endocrine and metabolic components profoundly influence female fertility, including hormone production as well as the development and ovulation of healthy oocytes. The role of PPARG in these events is two-fold. PPARG activation of transcription has outcomes operating both directly within the ovarian structure itself, and also indirectly through influences on other tissue systems such as the adipose tissue and immune cells (Figure 3). In this way, PPARG controls key signals regulating the capacity for normal reproduction. As PPARG is able, and required, to regulate many of these actions, it is important that the roles of PPARG be carefully considered as new concepts develop regarding the effects of dietary supplements such as PUFAs, which are PPARG ligands, and the consequences of increased immunological activation, such as occurs during obesity. As the health crisis surrounding the obesity epidemic widens to include the damaging effects on female fertility, it is important to remember the systemic implications of metabolism and immune regulation on female fertility, and to consider the role of PPARG in coordinating these contributions. Tremendous opportunity exists for those interested in elucidating further the exciting interactions between PPARG and female fertility. Publication of the most extensive list to date of all genes containing potential PPREs in their promoter regions [207] will provide a valuable tool for such research, as many identified genes have known functions within the context of ovarian physiology and pathology, in addition to characterized roles in other tissues, including macrophages and adipose tissue.

## Figures and Tables

**Table 1:** *Phenotypes and reproductive effects associated with PPARG mutations in mice and humans*. Abbreviations used: ART: artificial reproductive technology; BAT: brown adipose tissue; BMI: body mass index; HbA(1C): haemoglobin A1C; KO: knock-out; PCOS: polycystic ovary syndrome; T2DM: Type 2 Diabetes Mellitus; TG: Triglycerides; WAT: white adipose tissue.

Species	Genetic Abberation	Outcome	Effect on female fertility	Reference
Mouse	Global PPARG^−/−^	Neonatal death	—	[23]
	Global PPARG^−/+^	Improved insulin sensitivity	Fertile	[39]
	Mammary, epithelium, ovary, B- and T-cell null	Ovarian dysfunction and abrogated mammary development	30% of animals completely infertile, remainder had delayed conception, reduced litter size	[7]
	PPARG^hyp/hyp^: WAT BAT, liver, and muscle null.	Normal birthweight but subsequent growth retardation, lipodystrophy, hyperlipideamia, and mild glucose intolerance	Heterozygote matings produce normal sized litters, but homozygote matings result in reduced litter size.	[40]

Human	Pro12Ala (34C > G), PPARG2 only.	Ala allele ↓PPREs affinity, ↓PPARG transactivation. ↑Insulin sensitivity in some studies, conflicting reports on association with BMI.	Possible relationship with PCOS. In wider, non-PCOS population Ala allele associated with ↓testosterone production	[41–43]
	Pro115Gln (344G > T), PPARG2 only.	Constitutively activated PPARG, ↑adipocyte differentiation. Severe obesity, 3/4 subjects T2DM.	Fertility not assessed.	[44]
	His447His (1431C > T)	T allele may increase adipocyte differentiation. Presence of T allele associated with ↑BMI, and insulin sensitivity.	T allele more common in PCOS compared to BMI-matched controls. T allele associated with ↓testosterone.	[45, 43]
	Pro467Leu (1647C > T)	Mutation in LBD, ↓coactivator recruitment and downstream transactivation. ↓Basal gene activity. Lipodystrophy but normal BMI, severe insulin resistance and hypertension. One carrier (from 4) responsive to rosiglitazone therapy.	Oligomenorrhoea and hirtsutism, required ART for 1st pregnancy, complicated by pre-eclampsia and induced labour. 2nd pregnancy spontaneously conceived, with pre-eclampia, preterm emergency caesarean, and neonatal infant death.	[46, 47]
	Val290Met (1115G > A)	Mutation affects LBD, profound blockage of transcriptional activation. Similar phenotype to P467L. Unresponive to rosiglitazone therapy.	Primary amenorrhoea, hirsutism, acanthosis nigricans, and hypertension.	[46, 47]
	Phe388Leu (1164T > A)	↓PPARG-ligand binding, ↓basal transcriptional activity. Lipodystrophic and hypertensive with ↑TG. Hyperinsulinemic, later T2DM.	Irregular menses, and bilateral polycystic ovaries treated with salpingo-oopherectomy. Prior to this carried two pregnancies.	[48]
	Arg397Cys (1273C > T)	Mutation in LBD, unknown effect on PPARG function. Lipodystrophic, ↑TG and T2DM.	Hirsutism but no other indications of hyperandrogenism. Delayed menarche, but regular menses.	[49]

**Table 2:** *Summary of reports published within the past 2 years on the use of PPARG activating agents for reproductive symptoms*. Abbreviations used: AUC, area under the curve; BMI body mass index; CC clomiphene citrate; DHEA-S dehydroepiandrosterone sulfate; E2, estradiol; FAI, free androgen index; FSH, follicle-stimulating hormone; GnRH, gonadotropin releasing hormone; HbA(1C), haemoglobin A1C; HDL-C, high density lipoprotein-cholesterol; HOMA, homeostasis model of assessment for insulin sensitivity; IGF1 insulin-like growth factor 1; IGFBP-1/3, insulin-like growth factor binding protein 1 or 3; LDL-C, low density lipoprotein-cholesterol; LH, luteinizing hormone; OGTT, oral glucose tolerance test; PCOS, polycystic ovary syndrome; QUICKI, quantitative insulin-sensitivity check index; SHBG, sex hormone binding globulin; T, testosterone; WHR, waist to hip ratio.

Reference:	Rautio et al. [75] and Rautio et al. [76]
Patient profile:	Overweight but not obese PCOS (*n* = 30)
PPAR agonist:	Rosiglitazone (4 mg once daily for 2 weeks then 4 mg twice daily for 16 weeks)
Metabolic outcomes:	Serum C-reactive protein levels, leukocyte count, and alanine aminotransferase enzyme activity decreased, but lipid and blood pressure did not change. Glucose tolerance and peripheral insulin response normalized in the rosiglitazone group.
Reproductive outcomes:	Rosiglitazone improved menstrual cyclicity, SHBG levels; and decreased serum levels of androstenedione, 17-hydroxyprogesterone (17-OHP), DHEA and DHEA-S.

Reference:	Rouzi and Ardawi [77]
Patient profile:	Obese PCOS (*n* = 12)
PPAR agonist:	Rosiglitazone (4 mg twice daily for 3 cycles, CC administered for 5 days starting 3 days after rosiglitazone initiated)
Metabolic outcomes:	No changes in fasting plasma glucose or HbA(1C) or IGFBP-3 values. Fasting serum insulin, DHEA-S, androstenedione, and IGF-1 levels decreased significantly and IGFBP-1 exhibited significant increases.
Reproductive outcomes:	Total-T, free-T, LH, and SHBG decreased. Follicular development and ovulation rate increased, trend for increased pregnancy rate in group receiving short-term administration of rosiglitazone compared to matched control receiving metformin.

Reference:	Mitkov et al. [78]
Patient profile:	Obese, insulin resistant PCOS (*n* = 15)
PPAR agonist:	Rosiglitazone (4 mg/day for 12 weeks)
Metabolic outcomes:	Hyperinsulinemia and insulin resistance normalized.
Reproductive outcomes:	Total-T and FAI profile tended to normalise. Number of women with oligomenorrhea was reduced by 67%

Reference:	Cataldo et al. [79]
Patient profile:	Insulin resistant PCOS (*n* = 11–16/group)
PPAR agonist:	Rosiglitazone (2, 4 or 8 mg/day for 12 weeks)
Metabolic outcomes:	Steady state plasma glucose declined and hyperinsulinemia fell in a dose-dependent manner.
Reproductive outcomes:	Serum LH, total-T, and free-T were unchanged; SHBG increased. Ovulation occurred in 55%, without significant dose dependence. Before and during treatment, ovulators on rosiglitazone had lower circulating insulin and free-T and higher SHBG than nonovulators.

Reference:	Lemay et al. [80]
Patient profile:	Overweight, insulin resistant PCOS (*n* = 15)
PPAR agonist:	Rosiglitazone (4 mg/day for 6 months)
Metabolic outcomes:	Plasma insulin, insulin resistance indices and insulin AUC in response to OGTT all decreased compared to controls receiving antiandrogenic estrogen-progestin. Effect on lipids was limited.
Reproductive outcomes:	No significant effect on androgens or hirsutism.

Reference:	Garmes et al. [81]
Patient profile:	Obese insulin resistant PCOS (*n* = 15)
PPAR agonist:	Pioglitazone (30 mg/day for 8 weeks)
Metabolic outcomes:	Insulin response to OGTT significantly decreased.
Reproductive outcomes:	Total-T and free-T levels decreased, SHBG increased, and LH response to GnRH stimulation decreased.

Reference:	Yilmaz et al. [82–84]
Patient profile:	Obese or lean PCOS (*n* = 20 obese, *n* = 20 lean)
PPAR agonist:	Rosiglitazone (4 mg/day for 12 weeks)
Metabolic outcomes:	Indices of oxidative stress improved. HOMA, insulin AUC, fasting insulin and C-peptide levels decreased significantly. Glucose/insulin ratio and BMI increased

Reference:	Rautio et al. [75] and Rautio et al. [76]
Patient profile:	Overweight but not obese PCOS (*n* = 30)
PPAR agonist:	Rosiglitazone (4mg once daily for 2 weeks then 4 mg twice daily for 16 weeks)
Metabolic outcomes:	Serum C-reactive protein levels, leukocyte count, and alanine aminotransferase enzyme activity decreased, but lipid and blood pressure did not change. Glucose tolerance and peripheral insulin response normalized in the rosiglitazone group.
Reproductive outcomes:	Rosiglitazone improved menstrual cyclicity, SHBG levels; and decreased serum levels of androstenedione, 17-hydroxyprogesterone (17-OHP), DHEA and DHEA-S.

Reproductive outcomes:	Seurm levels of free-T, androstenedione, and DHEA-S decreased significantly. Menstrual disturbances improved in 61.5% of lean and 53.8% of obese patients. In a second cohort of patients, menstrual cycles became regular in 87.8%.

Reference:	Tarkun et al. [85]
Patient profile:	Young, lean PCOS (*n* = 31)
PPAR agonist:	Rosiglitazone (4 mg/day for 12 months)
Metabolic outcomes:	Fasting insulin and insulin resistance indices significantly improved. No changes in BMI, waist circumference, serum total cholesterol, or LDL-C. Serum C-reactive protein levels decreased; and endothelium-dependent vascular responses improved.
Reproductive outcomes:	Significant decreases in serum T, although no change in FSH and LH levels. Hirsutism score decreased significantly after treatment. 77.4% of women reverted to regular menstrual cycles. Levels of SHBG increased significantly after treatment.

Reference:	Dereli et al. [86]
Patient profile:	Nonobese PCOS (*n* = 20/group)
PPAR agonist:	Rosiglitazone (2 mg/day or 4 mg/day for 8 months)
Metabolic outcomes:	75% of women in the 2 mg group and 95% in the 4 mg group achieved normal glucose tolerance. Improved insulin resistance in a dose-related fashion, without adverse events or liver enzyme elevations.
Reproductive outcomes:	Decreased free-T levels were better in the 4 mg group than the 2 mg group, and 70% of women in the 2 mg group and 85% of women in the 4 mg group achieved ovulatory menses.

Reference:	Mehta et al. [87]
Patient profile:	Obese PCOS (*n* = 9)
PPAR agonist:	Pioglitazone (45 mg/day for 20 weeks)
Metabolic outcomes:	Significant improvement in insulin sensitivity
Reproductive outcomes:	LH levels, LH pulse frequency and amplitude, as well as gonadotropin responses to GnRH were not influenced.

Reference:	Ortega-González et al. [88]
Patient profile:	Obese, insulin resistant PCOS (*n* = 25)
PPAR agonist:	Pioglitazone (30 mg/day for 6 months)
Metabolic outcomes:	Body weight, BMI, and WHR increased significantly. Fasting insulin and insulin AUC during a 2-h OGTT decreased. Insulin resistance decreased and insulin sensitivity increased after treatment with either pioglitazone or metformin received by control group.
Reproductive outcomes:	Hirsutism, free-T and androstenedione declined to a similar extent after treatment with either drug. Treatment with both drugs was associated with the occurrence of pregnancy

Reference:	Sepilian and Nagamani [89]
Patient profile:	Obese insulin resistant PCOS (*n* = 12)
PPAR agonist:	Rosiglitazone (4 mg/day for 6 months)
Metabolic outcomes:	Fasting insulin, insulin AUC, fasting glucose, and glucose AUC significantly decreased. No significant change in BMI
Reproductive outcomes:	Both total-T, free-T and DHEA-S levels decreased significantly. No significant change in LH levels. Levels of SHBG increased significantly after treatment, 91.7% of women reverted to regular ovulatory cycles during the treatment period
